# Polymethyl Methacrylate-like Photopolymer Resin with Titanium Metal Nanoparticles Is a Promising Material for Biomedical Applications

**DOI:** 10.3390/polym17131830

**Published:** 2025-06-30

**Authors:** Dmitriy E. Burmistrov, Dmitriy A. Serov, Ilya V. Baimler, Ann V. Gritsaeva, Pavel Chapala, Aleksandr V. Simakin, Maxim E. Astashev, Ekaterina E. Karmanova, Mikhail V. Dubinin, Guliya R. Nizameeva, Shamil Z. Validov, Fatikh M. Yanbaev, Oleg G. Synyashin, Sergey V. Gudkov

**Affiliations:** 1 Prokhorov General Physics Institute of the Russian Academy of Sciences, Vavilov Str. 38, 119991 Moscow, Russia; dmitriiburmistroff@gmail.com (D.E.B.); dmitriy_serov_91@mail.ru (D.A.S.); ilyabaymler@yandex.ru (I.V.B.); anngritsaeva@mail.ru (A.V.G.); avsimakin@gmail.com (A.V.S.); astashev@yandex.ru (M.E.A.); dubinin1989@gmail.com (M.V.D.); 2 HARZ Labs LLC, Silikatnaya Str. 51AC6, 141006 Mytischi, Russia; p.chapala@harzlabs.com; 3 Federal Research Center “Pushchino Scientific Center for Biological Research of the Russian Academy of Sciences”, Institute of Cell Biophysics of the Russian Academy of Sciences, 3 Institutskaya St., 142290 Pushchino, Russia; silisti@bk.ru; 4 Department of Biochemistry, Cell Biology and Microbiology, Mari State University, pl. Lenina 1, 424001 Yoshkar-Ola, Russia; 5 Department of Physics, Kazan National Research Technological University, 68, K. Marx str., 420015 Kazan, Russia; guliya.riv@gmail.com; 6 Federal Research Center Kazan Scientific Center of the Russian Academy of Sciences, ul. Lobachevskogo 2/31, Tatarstan, 420088 Kazan, Russia; sh.validov@knc.ru (S.Z.V.); f.yanbayev@knc.ru (F.M.Y.); oleg@iopc.ru (O.G.S.)

**Keywords:** titanium nanoparticles, polymethyl methacrylate-like photopolymer, photopolymer resin, antibacterial properties

## Abstract

New materials for additive manufacturing are currently being actively studied, which both have the necessary physicochemical properties and are safe for the environment and living organisms. We have proposed a simple process for the production of composite materials based on a transparent polymethyl methacrylate-like photopolymer resin modified with metallic titanium nanoparticles. Standardized plate samples were printed from the obtained modified photopolymer resins using mask stereolithography with an LED light source array (MSLA), and their mechanical properties were evaluated. Plates were also printed, for which the surface topology, distribution of nanoparticles in the polymer matrix, chemical structure, optical properties, chemical structure, and optical properties were characterized. In the context of the impact on biological systems, the ability of materials to enhance the formation of ROS and affect the main biomacromolecules was demonstrated. At the same time, the developed composite materials inhibit the growth of *E. coli* bacterial cells, and the bactericidal effect of the surfaces of the obtained materials was shown. Despite the significant antibacterial properties of the synthesized materials, no negative impact on the growth and development of adhesive cultures of eukaryotic cells in vitro was detected.

## 1. Introduction

Materials based on modified methacrylates are widely used and are in demand in modern photopolymer 3D printing technologies [1,2,3,4]. Polymethacrylates are a group of thermoplastic polymers, derivatives of esters of acrylic, methacrylic, or cyanoacrylic acids [5]. These polymers have found very wide application in various fields, including dentistry (printing of tooth models, dental products, temporary crowns) [6], medical prosthetics [7], optical engineering (production of optical products, lenses, and light guides) [8,9,10,11], jewelry industry (modeling and prototyping of products), chemical industry and engineering [12,13], and, in general, are a popular structural material, including for the creation of high-precision products [14,15,16,17]. It is noteworthy that a number of polymethacrylates, in particular polymethyl methacrylate (PMMA), have been approved by the Food and Drug Administration (FDA) for use in a number of medical devices [18], including intraocular lenses and bone cement, which emphasizes its safety for the environment and effectiveness in clinical applications [7,18,19]. Despite the prevalence of polymethacrylate materials, a number of significant drawbacks have been identified that require additional processing or the introduction of additional functionalizing additives: low impact resistance, limited heat resistance, chemical vulnerability, hygroscopicity, the need for additional multi-stage post-processing of products, and susceptibility to biodegradation and bacterial contamination [20,21,22]. One of the widely studied methods for improving the physicochemical and biological properties of polymer matrices in recent years is modification with nanoparticles [23,24,25]. In terms of physicochemical properties, the addition of metal and metal oxide NPs to polymer matrices has been reported to significantly increase tensile strength [26,27], flexural modulus [28], hardness [29,30], decrease glass transition temperature and heat resistance [31], and increase UV resistance [32,33]. In the context of biological properties, significant antibacterial and antifungal effects of metal and metal oxide nanoparticles have been shown [34,35,36,37,38,39,40,41].

Currently, a fairly large number of metals are used as nanosized dopants in polymers [42]. This work uses titanium nanoparticles. Titanium has a number of advantages over many other metals [43]. Titanium is one of the lightest metals, its density is approximately 4.5 g/cm^3^. For comparison, the density of steel is about 7.8 g/cm^3^, copper—about 8.9 g/cm^3^, silver—about 10.5 g/cm^3^, gold—19.3 g/cm^3^. Only aluminum is a lighter metal (2.7 g/cm^3^); however, aluminum quickly oxidizes in the presence of oxygen and forms aluminum oxide [44]. Pure titanium is non-magnetic, just like photopolymer resin, which is certainly also an advantage [45]. Titanium is also resistant to corrosion, resistant to most acids, with the exception of concentrated sulfuric and fluoric acids, and to many alkalis [46]. Unlike titanium oxide, metallic titanium does not have photocatalytic properties, which in some ways facilitates its use in biomedicine [47].

In view of the rapidly developing additive manufacturing industry and the emergence of new high-precision and biosafe materials for 3D modeling and design, studies aimed at obtaining photocurable resins with the addition of biologically active additives are of considerable interest [48,49]. It is well known that titanium nanoparticles are an antimicrobial agent acting as an effective functionalizing additive in various polymeric materials [50,51,52,53]. The aim of this work is to obtain a series of new composite materials for photopolymer printing based on polymethyl methacrylate-like photopolymer resin with the addition of Ti-NPs and to study the physicochemical, biological, and operational properties.

## 2. Materials and Methods

### 2.1. Synthesis and Characterization of Ti-NPs; Preparation of PMMA/Ti-NPs Composite Material

Ti-NPs were synthesized by laser ablation of a solid polished titanium metal target (99.99%) placed in chemically pure acetone (Komponent-Reaktiv, Moscow, Russia). In the experiment, a second-harmonic Nd:YaG laser (λ = 532 nm, f = 1 kHz, τ = 4 ns, Ep = 1.5 mJ) was used as a radiation source. The volume of the working fluid was 30 mL. The details of the laser ablation procedures were described previously [54]. A Malvern Zetasizer Ultra (Malvern Panalytical Ltd., Malvern, UK) was used to characterize the size, concentration, and ζ-potential of Ti-NPs dispersions in acetone. A 10 × 10 mm quartz cuvette and a ‘Dip’ Cell ZEN1002 electrode (Malvern Panalytical Ltd., UK) were used for the measurements. The UV-Vis absorption spectrum of the Ti-NPs colloidal solution was obtained using a CINTRA 4040 (GBC Scientific Equipment Pty Ltd., Victoria, Australia). The morphology of the Ti-NPs was assessed using a Libra 200 FE HR transmission electron microscope (TEM) (Carl Zeiss, Jena, Germany). The colloidal Ti-NPs solution obtained by laser ablation was homogeneously distributed in a photopolymer resin based on methacrylic monomers and oligomers Clear PRO (Harz Labs, Mytishchi, Russia) using mechanical and ultrasonic stirring to final Ti-NPs concentrations of 0.001, 0.01, and 0.1 wt%. The colloid of nanoparticles was introduced into the thickness of the resin using an automatic microdispenser dropwise, followed by mixing and holding. The photopolymer resin used is a transparent liquid with a viscosity of 1000 ± 200 mPa×s. The products printed from the resin used are highly transparent, precise, chemically resistant, and resistant to autoclaving. This material meets the requirements of ISO 10993 [55] and is an approved material for medical purposes in the Russian Federation (registration certificate for a medical device No. RZN 2020/12007). The resulting mixture was poured into a measuring cup and subjected to a stirring cycle twice: 5 min of treatment on an MS 3 digital shaker (2500 rpm, amplitude 4.5 mm) (IKA, Wilmington, NC, USA) and 5 min of ultrasound treatment in a PS-20A bath (Digital China Group Co., Ltd., Shenzhen, Guangdong, China) (40 kHz, 22 °C). Before printing, the modified resin was kept for at least 20 h. The obtained resin samples were stored in dark glass bottles V = 500 mL, sealed with hermetically sealed screw caps in a place isolated from direct light.

### 2.2. Additive Fabrication of PMMA/Ti-NPs Composite Samples

A Saturn 3 Ultra 12K MSLA printer (Elegoo, Shenzhen, China) was used to print from resins containing Ti-NPs. Before starting the printing, a pre-prepared resin containing Ti-NPs at a concentration of 0.001, 0.01 or 0.1 wt% was poured into a clean bath. Clear Pro photopolymer resin (Harz Labs, Mytishchi, Russia) without the addition of nanoparticles was used to obtain control samples. For each group of studied Ti-NPs concentrations in MA, the following printed samples were used: ISO 179-1:2010 Type 1 [56] and ISO 527-2:2012 [57] Type 1A, as well as round plates ⌀16 mm and 0.5 mm thick for physicochemical and biological studies (n = 3 for each group). After the photopolymer printing cycle, the samples were separated from the platform, washed in absolute isopropyl alcohol (99.9%) for 6 min using a washing tank placed on the magnetic platform of the UW-02 3D product cleaning and curing device (Creality3D, Shenzhen, China). Then the samples were additionally processed in an ultrasonic bath filled with absolute isopropyl alcohol for 6 min, dried at room temperature for 10 min, treated with glycerin, and irradiated for 30 min in a 3D product curing chamber with a UW-02 rotating platform (Creality3D, China). After curing, the samples were re-processed in an ultrasonic bath filled with isopropyl alcohol for 6 min, dried at room temperature for 10 min, then placed in a dry-heat oven for 30 min at 80 °C. Samples ready for further research were stored in closed Petri dishes under normal conditions.

### 2.3. Characterization of PMMA/Ti-NPs Material Samples

The surface topology of the printed products was assessed using an atomic force microscope (NT-MDT LLC, Zelenograd, Russia), which allows studying the surface micro- and nanostructure in non-contact and semi-contact modes. To study the interaction of Ti-NPs with the polymer matrix of polymethacrylate, an IR-8000 FTIR spectrometer (SAS LLC, Krasnoyarsk, Russia) with a ZnSe Sealed Flat Plate (Pike Technologies, Fitchburg, WI, USA) was used. The optical properties of the obtained samples in the visible and UV wavelength ranges were studied using a Cintra 4040 dual-beam UV/Vis spectrometer (GBC Scientific Equipment Pty Ltd., Victoria, Australia). An MIM-321 modulation interference microscope (Amphora laboratories, Moscow, Russia) was used to assess the distribution of nanoparticles in the polymethacrylate matrix. The mechanical properties (tensile and bending tests) of the printed samples Type 1A, Type B (ISO 527-2:2012) were evaluated using a universal testing machine WDW-5S (Hongtuo, Binzhou, China). The bending tests were carried out in accordance with ASTM D790 [58], the tensile tests were carried out in accordance with ASTM D638-22 [59].

### 2.4. Quantitative Assessment of Generated ROS in Aqueous Solutions (H_2_O_2_ and ^•^OH)

Composite material samples were used in the form of 10 × 10 × 0.5 mm plates. The samples were incubated in 10 mL of deionized water at 40 °C for 180 min. The concentration of hydrogen peroxide was measured immediately after sample incubation. The concentration of hydrogen peroxide formed in aqueous solutions was determined by chemiluminescence using the luminol-p-iodophenol–horseradish peroxidase system. The chemiluminescent signal was recorded using a highly sensitive chemiluminometer “Biotox-7A-USE” (Engineering Center—Ecology, Moscow, Russia). The samples of the studied composite material were placed in polypropylene vials (Beckman, CA, USA), to which 1 mL of the “counting solution” prepared immediately before the measurement was added. The composition of the solution included 1 mM Tris-HCl buffer (pH 8.5), 50 μM p-iodophenol, 50 μM luminol, and 10 nM horseradish peroxidase. All experimental details have been described previously [60]. The method is characterized by high sensitivity, ensuring the detection of hydrogen peroxide at concentration of less than 0.1 nM. Samples of composite materials were used in the form of plates measuring 10 × 10 × 0.5 mm. The samples were incubated in 10 mL at 80 °C for 120 min. The amount of hydroxyl radicals was measured immediately after sample incubation. A fluorimetric method based on the interaction of hydroxyl radicals with coumarin-3-carboxylic acid (CCA) was used for the quantitative determination of hydroxyl radicals. An aqueous solution of CCA 0.5 mM was used as a reaction medium. The reaction resulted in the formation of fluorescent product—7-hydroxycoumarin-3-carboxylic acid (7-OH-CCA). The fluorescence intensity of 7-OH-CCA was recorded using a JASCO 8300 spectrofluorimeter (JASCO, Tokyo, Japan) at an excitation wavelength of 400 nm and an emission wavelength of 450 nm.

### 2.5. Quantitative Determination of 8-Oxoguanine and Long-Lived Reactive Proteins Species (LRPS)

Quantitative determination of 8-oxoguanine (8-oxoGua) in DNA samples was performed using a non-competitive enzyme-linked immunosorbent assay (ELISA) with monoclonal antibodies specific for 8-oxoGua [61]. DNA obtained from the samples was diluted to a concentration of 350 μg/mL. For denaturation, the samples were heated in a water bath for 5 min, then cooled on ice for 3–4 min. Aliquots of 42 μL were applied to the wells of an ELISA plate. DNA was immobilized by passive adsorption at 80 °C for 3 h until the solution was completely dry. Blocking of non-specific adsorption sites was performed by adding 300 μL of blocking solution containing 1% non-fat dry milk dissolved in 0.15 M Tris-HCl buffer (pH 8.7) with the addition of 0.15 M NaCl to each well. The plates were incubated at room temperature overnight (14–18 h). To form the antigen–antibody complex, 100 μL of the working solution of primary antibodies (monoclonal antibodies against 8-OG, diluted 1:2000 in blocking solution) was added and the plates were incubated at 37 °C for 3 h. After this, the wells were washed twice with 300 μL of 50 mM Tris-HCl buffer (pH 8.7) containing 0.15 M NaCl and 0.1% Triton X-100, with incubation for 20 min. Next, 80 μL of blocking solution containing secondary antibodies (anti-mouse immunoglobulins conjugated with horseradish peroxidase, dilution 1:1000) were added to the wells and incubated at 37 °C for 1.5 h. After washing three times as described above, 100 μL of chromogenic substrate containing 18.2 mM ABTS and 2.6 mM H_2_O_2_ in 75 mM citrate buffer (pH 4.2) were added to each well. The reaction was stopped by adding 100 μL of 1.5 mM NaN2 in 0.1 M citrate buffer (pH 4.3) after visible staining was achieved. Optical density was measured at a wavelength of 405 nm using a Feyond-A400 plate photometer (Allsheng, Hangzhou, China).

LRPS concentration was estimated by recording chemiluminescence of protein solutions after incubation at 40 °C for 2 h. Composite material samples were used as 10 × 10 × 0.5 mm plates. The samples were incubated in 10 mL of 0.1% BSA aqueous colloid at 40 °C for 120 min. After incubation, the samples were kept at room temperature in the dark for 30 min. Measurements were performed in 20 mL polypropylene vials (Beckman, Brea, CA, USA) under complete darkness at room temperature. Highly sensitive Biotox-7A chemiluminometer (Engineering Center—Ecology, Moscow, Russia) was used as an instrument. Bovine serum albumin (BSA) solutions that were not exposed to heating were used as a control.

### 2.6. E. coli Growth Curves

Previously printed samples of PMMA/Ti-NPs composite material plates were cut into 10 × 10 mm pieces using scissors and placed into the wells of 24-well plates (TPP, Trasadingen, Switzerland) using tweezers. The wells of the plates were filled with 1 mL of 70% ethyl alcohol solution using a micropipette, then the alcohol was removed, and the plates were left in a laminar flow hood with a UV lamp turned on for 40 min. Next, a bacterial suspension was prepared: 10 mL of sterile LB microbiological broth (DiaM, Moscow, Russia) was placed in a sterile glass bottle V = 50 mL (MiniMed, Suponevo, Russia), and part of the *E. coli* colony was transferred to the bottle with the broth using a microbiological loop burned in an alcohol lamp flame. The vial was tightly closed with a lid and placed overnight in an ES-20 shaker-incubator (Biosan, Riga, Latvia) (37 °C, 230 rpm). Before the experiment to assess the growth rate of bacteria on the surface of new composite polymer materials, the overnight broth culture of *E. coli* was diluted 1000 times in sterile microbiological broth LB (DiaM, Moscow, Russia) (~106 cells/mL), thoroughly mixed using a V-1 plus vortex (Biosan, Riga, Latvia). Then, 1000 μL of inoculated broth was added to each well with the test sample of the test material under a laminar cabinet. The plate without a lid was placed in the holder of the Feyond-A400 plate photometer (Allsheng, Hangzhou, China), which allows for long-term experiments due to the built-in thermostatting and shaking functions. Based on the obtained data, growth curves of bacterial cells were constructed and the influence of the studied materials on the growth kinetics of bacterial cultures was analyzed.

### 2.7. Evaluation of Antibacterial Activity by Flow Cytofluorometry

For additional microbiological studies, flow cytometry was used using a Longcyte cytofluorimeter (Challenbio, Beijing, China). Sample preparation and bacterial cultivation in the presence of PMMA/Ti-NPs composite material samples was carried out in the same manner as described above. After completion of cultivation, 1 mL of phosphate-buffered saline (PBS) (Sigma-Aldrich, St. Louis, MO, USA) containing 4 μM propidium iodide (PI) (Lumiprobe, Westminster, MD, USA) was added to each sample in of the wells of a 24-well plate. After binding to DNA, the PI dye emits in the orange-red channel with absorption maximum at 535 nm and emission maximum at 617 nm. The quantum yield of the complex with DNA is 20–30 times higher than the yield of the free dye. The plate with the added dye was incubated for 60 min in the dark. Then the samples were resuspended and transferred to clean Expell Microcentrifuge Tubes V = 1.5 mL (Capp, Nordhausen, Germany), which were placed in the device stand.

### 2.8. In Vitro Cytotoxicity Assessment

The printed PMMA/Ti-NPs polymer composite samples were tested against HSF cell cultures (ATCC #PCS-201-012). The cells were cultured and subcultured according to standard protocols in freshly prepared DMEM/F12 culture medium supplemented with 10% FBS, 2 mM L-glutamine, 25 U/mL penicillin, and 25 μg/mL streptomycin (all PanEco, Moscow, Russia). On the day of the experiment, the cell suspension 105/200 μL/glass was applied to the surfaces of round ⌀25 mm cover glasses (MiniMed, Russia), pre-sterilized at 180 °C for 2 h, placed on the bottom of the wells of a 6-well culture plate (TPP, Trasadingen, Switzerland). Drops of cell suspension on glass slides were incubated for 30 min in a S-Bt Smart Biotherm CO_2_ incubator (Biosan, Riga, Latvia) (37 °C, 5% CO_2_) for adhesion to the glass surface. Then, 1800 μL of warm ready-made culture medium was added to each well and one sample of the tested polymer material was immersed. After 72 h of in vitro cultivation, the samples were removed from the plate wells with tweezers, the cells were analyzed through a light microscope and then vital staining was carried out with fluorescent dyes Hoechst 33342, Rhodamine and PI (all Lumiprobe, Westminster, MD, USA), allowing visualization of nuclei, mitochondria (cytoplasm contrasting) of cells and non-viable cells, respectively. The DMI 4000B system (Leica, Wetzlar, Germany) was used for visualization and registration of microphotographs. The resulting images were analyzed using Fiji ImageJ software 2.14.0. For each sample, five independent measurements were performed. All experimental details have been described previously [62].

### 2.9. Statistical Data Analysis and Visualization

Experimental data processing and statistical analysis were performed using GraphPad Prism 8.3.0 software. Results are presented as mean values ± standard error of the mean. Results from at least three independent experiments were used for averaging. Significance level *p* < 0.05.

## 3. Results

**Figure 1:**
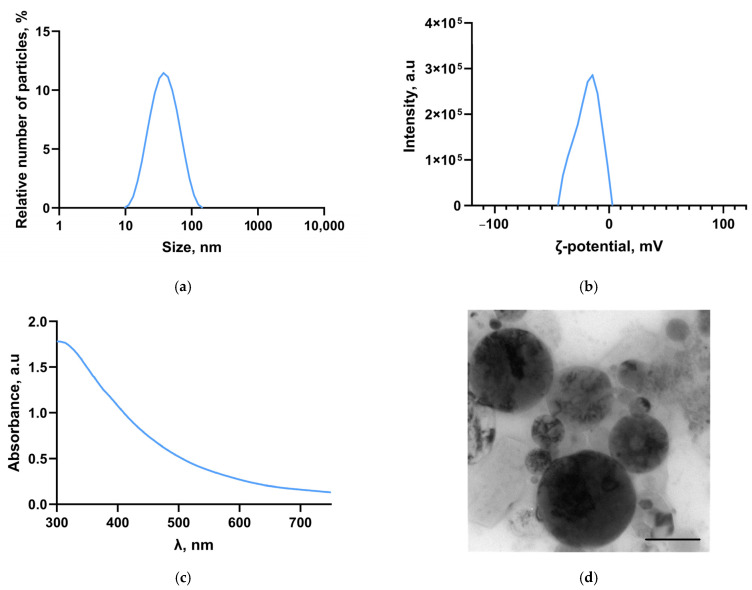
Characteristics of the physicochemical properties of Ti-NPs obtained by laser ablation. Nanoparticle size distribution obtained by dynamic light scattering (**a**). Distribution of the ζ-potential of Ti-NPs in the colloid (**b**). UV-Vis absorption spectrum of the Ti-NPs colloid (**c**). TEM image of Ti-NPs (scale bar is 30 nm) (**d**).

The size distribution of titanium nanoparticles was studied using dynamic light scattering (Figure 1a). It was shown that the obtained distribution is monomodal; the maximum particle size distribution has an abscissa of about 35 nm. The half-width of the distribution at half-maximum is about 60 nm. The distribution of the electrokinetic potential of Ti-NPs in the colloid was studied (Figure 1b). It was shown that the maximum of the ζ-potential distribution falls at −16 mV. The half-width of the distribution at half-maximum is about 25 mV. The optical properties of the colloidal solution of Ti-NPs were studied in the range from 300 to 700 nm (Figure 1c). The highest light absorption is in the UV range. In other wavelength ranges, the absorption spectrum does not have any features; the absorption spectrum corresponds to Ti-NPs. Micrographs of Ti-NPs were obtained using TEM (Figure 1d). The nanoparticles, as can be seen from the obtained images, have a shape close to spherical. The size of the nanoparticles is generally consistent with the data obtained using dynamic light scattering. The photograph shows nanoparticles ranging in size from 15 to 50 nm, and the products of laser polymerization of acetone molecules are also visible in the form of a moire pattern.

**Figure 2:**
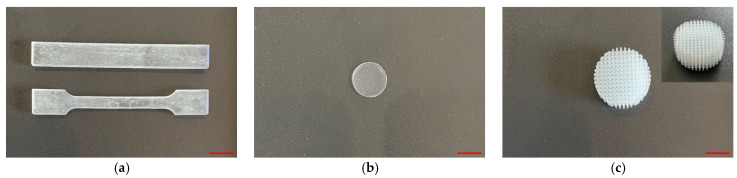
Photographs of products made of PMMA/Ti-NPs composite material. Photographs of printed PMMA/Ti-NPs samples for mechanical testing, point bending sample—ISO 179-1:2010 Type 1 (top) and for tensile testing according to ISO 527-2:2012 Type 1A (bottom) (**a**). Sample of printed plate for biological research ⌀16 mm (**b**). Demonstration sample with porous structure, printed using MLSA technology. The scale bar in the lower right corner of the photographs corresponds to 10 mm (**c**).

Nanoparticles were added to photopolymer resin (PMMA/Ti-NPs) at concentrations of 0.001, 0.01, and 0.1 wt%. To evaluate the strength properties of the material, 80 × 10 × 4 mm and 80 × 5 × 3 mm dog bone-shaped parts (ISO 179-1:2010 Type 1 and ISO 527-2:2012 Type 1A, respectively) were printed using MSLA 3D printing (Figure 2a). For further biological studies, thin ⌀16 mm plates with a thickness of 0.5 mm were also printed (Figure 2b). To study the printing accuracy of the obtained composite material, a porous 10 × 10 mm sample was printed with a cell size of 800 μm and edge sharpness in each cell of about 50 μm (Figure 2c).

**Figure 3:**
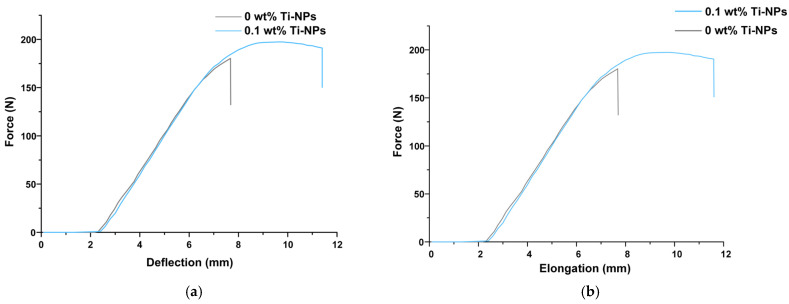
Stress–strain curves for PMMA/Ti-NPs samples containing 0.1 wt% Ti-NPs and the PMMA polymer control sample obtained in bending (**a**) and tensile (**b**) tests.

Using the standard ASTM D790 and D638-22 protocols, bending and tensile tests were performed. During the tests, it was found that for materials with the addition of 0.1 wt% Ti-NPs, more non-uniform plastic deformation was observed, while the PMMA material without Ti-NPs was deformed by rupture under tension (Figure 3a). In this case, the value of total elongation for PMMA/Ti-NPs composite materials exceeded the control values by ~30%. The tensile strength for samples of PMMA/Ti-NPs composite materials containing 0.1 wt% Ti-NPs was about 930 N, while for the PMMA polymer without the addition of Ti-NPs this parameter was 860 N. The tensile strengths were different for the PMMA/Ti-NPs composite and the polymer sample without nanoparticles, and were about 890 and 860 N, respectively. The flexural strength was also ~8% higher in the case of the composite compared to the PMMA polymer (Figure 3b).

**Figure 4:**
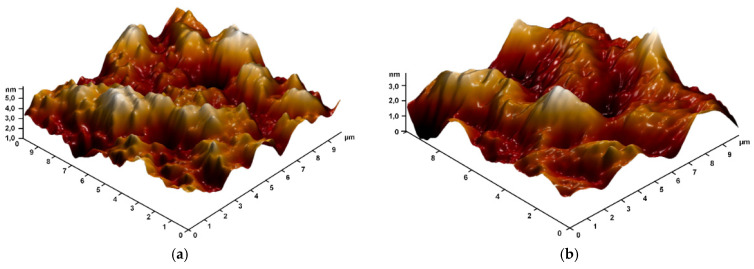
Reconstruction of the surface of printed material plate samples: surface of PMMA polymer without addition of Ti-NPs (control) (**a**); surface of PMMA/Ti-NPs composite material containing 0.1 wt% Ti-NPs (**b**).

For further studies, 0.5 mm thick round plates of PMMA/Ti-NPs materials were printed and polished under the same conditions. The surface topology of the obtained composite material samples was studied using atomic force microscopy (Figure 4). It was found that the surface of the material was uniform without significant defects (no cracks, cavities, voids, or craters from gas bubbles were detected). On the surface of the products after polishing, places were found in which the profile height did not exceed 5 nm. Thus, with the addition of titanium nanoparticles, the products can be subjected to precision optical polishing. Even at the highest concentration of nanoparticles, its effect on the surface profile of the products was not detected.

**Figure 5:**
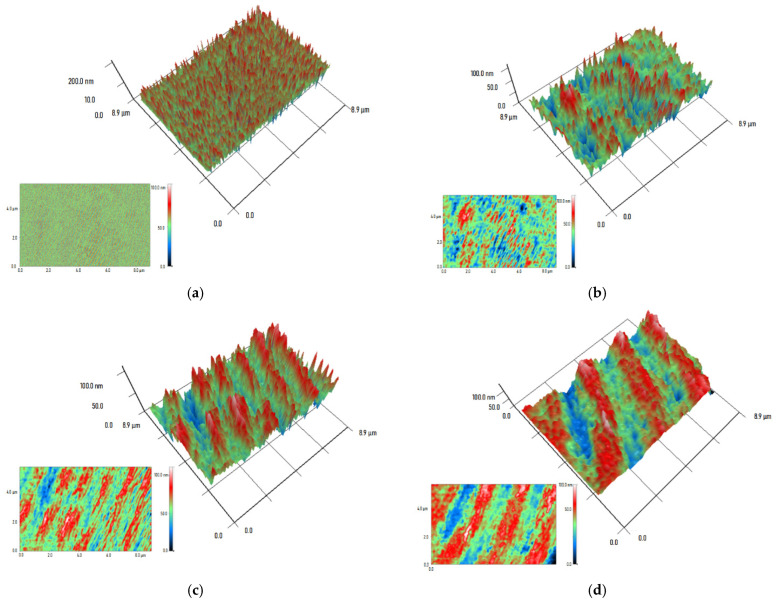
MIM micrographs and 3D reconstructions of PMMA-based composite material samples with the addition of Ti-NPs. PMMA without NPs (**a**), PMMA-based composite polymer materials with the addition of Ti-NPs at concentrations of 0.001 wt% (**b**), 0.01 wt% (**c**), and 0.1 wt% (**d**). Three-dimensional reconstructions of material sections measuring 8.9 × 8.9 μm and the primary data on which they were built are shown (insets at the bottom left). The color shows the phase difference in the transmitted radiation in nm (red is the maximum value, blue is the minimum).

To evaluate the distribution of nanoparticles in the polymer matrix, the modulation interference microscopy (MIM) method was used. The refractive index for metallic Ti is 2.61, while for pure PMMA it is 1.49, which makes these materials distinguishable using the MIM method. Pure MA polymer is devoid of pronounced optical inhomogeneities (Figure 5a), which indicates the integrity of the polymer matrix, and the absence of bubbles and other internal cavities inside the obtained products. The addition of Ti-NPs contributed to the appearance of optical inhomogeneities inside the composite material (Figure 5b–d). At the same time, the size of the inhomogeneities increased with an increase in the concentration of the introduced Ti-NPs. For PMMA/Ti-NPs 0.001% and PMMA/Ti-NPs 0.01%, the sizes of the inhomogeneities were ~0.5 × 0.2 μm and ~2.0 × 1.0 μm, respectively. In the case of PMMA/Ti-NPs 0.1%, the optical inhomogeneities were 1–2 μm wide and more than 8 μm long.

**Figure 6:**
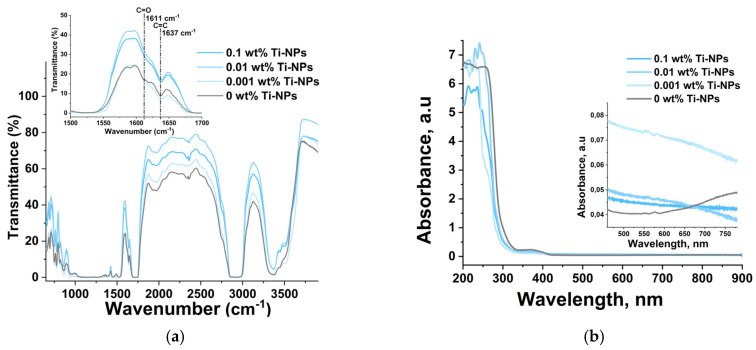
Study of spectral properties of samples printed from composite material based on methacrylate and titanium nanoparticles. FTIR transmission spectra of samples of PMMA/Ti-NPs composite material plates with different Ti-NPs content, inset—enlarged region of the spectrum with lines related to C=C double bonds (**a**). UV/Vis absorption spectra of samples of PMMA/Ti-NPs composite material plates with different Ti-NPs content, inset—enlarged part of the spectrum in the visible region (**b**).

To study the effect of Ti-NPs on the molecular structure of the PMMA matrix, IR and UV-Vis spectra were obtained. Figure 6a shows the averaged FTIR transmission spectra for the composite material samples. The insets in the figures show enlarged spectral regions with highlighted lines at 1611 and 1637 cm^−1^, related to the C=C double bonds. Polymerization of methacrylates occurs with the rupture of the carbon-carbon double bond in the methacrylate group. Therefore, the degree of polymerization of methacrylate resins can be estimated by the bands in the spectrum related to the C=C double bonds. It can be noted that almost all materials showed a noticeable decrease in the intensity of these bands in the spectra compared to PMMAwithout Ti-NPs. Figure 6b shows the UV-Vis absorption spectra of PMMA/Ti-NPs composite materials. The PMMAresin contains photoinitiators that allow photopolymerization of the material. The spectral region of 350–400 nm refers to the absorption of radiation by these photo-initiators. In general, it can be noted that the addition of Ti-NPs in all the concentrations considered did not significantly affect the optical properties of the polymethacrylate matrix in the visible range of the spectrum. All samples of PMMA/Ti-NPs composite material plates had a transparency range from 450 to 800 nm.

**Figure 7:**
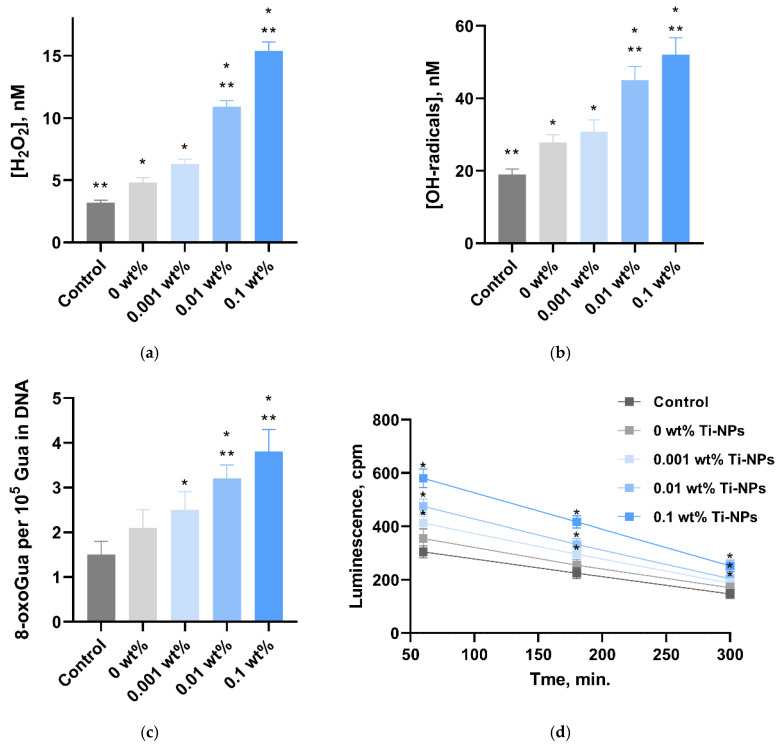
Study of the influence of samples printed from composite material based on methacrylate and titanium nanoparticles on the generation of active oxygen species and damage to biomacromolecules. Results of the influence of composite materials on the generation of hydrogen peroxide (H_2_O_2_) (**a**) and hydroxyl radicals (^•^OH) (**b**) in aqueous solutions (n = 3). Evaluation of the ability to cause oxidative damage to biomolecules in vitro: 8-oxoguanine in DNA (**c**) and long-lived reactive protein species (**d**). Data are presented as mean values ± SEM (n = 3). *—difference from control (*p* < 0.05), **—relative to samples of material not containing nanoparticles (0 wt%) (*p* < 0.05).

The ability of the synthesized materials to enhance the formation of reactive oxygen species (ROS) in aqueous solutions—hydrogen peroxide and hydroxyl radicals—was assessed (Figure 7a,b). The PMMA polymer samples without Ti-NPs contributed to the enhancement of the generation of H_2_O_2_ and OH-radicals, compared to the control (without sample). Compared to the control (3.2 ± 0.2 nM for H_2_O_2_ and 19.0 ± 1.5 nM for ^•^OH, respectively), all PMMA/Ti-NPs composite materials contributed to the enhancement of the generation of the considered ROS. The concentration of H_2_O_2_ was 6.3 ± 0.4, 10.9 ± 0.5, and 15.4 ± 0.7 nM when incubated with the PMMA/Ti-NPs samples of materials containing 0.001, 0.01, and 0.1 wt%, respectively. ^•^OH concentration was 30.8 ± 3.2, 45.0 ± 3.8, and 52.0 ± 4.7. It is noteworthy that for materials containing 0.01 and 0.1 wt% Ti-NPs, a more pronounced increase in ROS formation was revealed, compared to PMMA materials not containing Ti-NPs: ~2.2 and 3.2 times for H_2_O_2_ and ~1.6 and 1.87 times for ^•^OH, respectively.

The ability of the composite materials to induce oxidative DNA damage in vitro was studied (Figure 7c). The PMMA polymer samples without Tips did not affect the 8-oxoGua level in DNA compared to the control. The 8-oxoGua level in DNA did not differ between the samples without nanoparticles and the PMMA/Ti-NPs composite material containing 0.001 wt% Ti-NPs. The PMMA/Ti-NPs composite materials containing 0.01 and 0.1 wt% Ti-NPs contributed to an increase in the formation of 8-oxoguanines in DNA (3.2 ± 0.3 and 3.8 ± 0.5 per 10^5^ Gua in DNA, respectively, compared to both the control and the samples without nanoparticles).

The ability of composite materials to generate long-lived reactive protein species (LRPS) was studied (Figure 7d). It was shown that under control conditions, LRPS was formed with a half-life of about 5 h. In the control and PMMA samples not containing Ti-NPs, the generation of LRPS was quantitatively indistinguishable from each other. The half-life of LRPS induced by PMMA samples not containing Ti-NPs was also approximately 5 h. Composite PMMA/Ti-NPs materials containing 0.001–0.1 wt% Ti-NPs contributed to an increase in LRPS generation, while the half-life of LRPS remained unchanged and was equal to approximately 5 h. It should be noted that under the action of the composite material containing 0.1 wt% Ti-NPs, there was a twofold increase in ROS generation relative to the control occurred.

**Figure 8:**
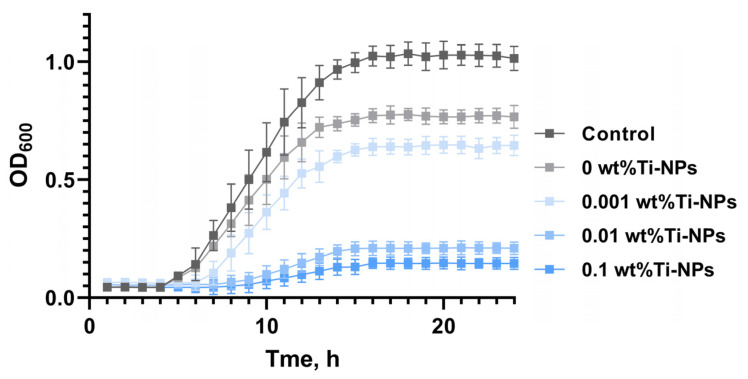
Growth curves of *E. coli* suspension cultures grown in the presence of composite material plate samples with different Ti-NPs content. Data are presented as mean values ± SEM (n = 3).

To evaluate the antibacterial properties of the synthesized samples of PMMA/Ti-NPs composite material plates, microbiological studies were conducted on suspension cultures. Figure 8 shows the growth curves of *E. coli* cells cultured in the presence of PMMA/Ti-NPs composite material plates for 24 h. According to the data obtained, the photopolymer material without the addition of Ti-NPs slightly changes the characteristics of the *E. coli* growth curve compared to the results for the control group. PMMA does not change the lag phase and does not significantly change the maximum bacterial density compared to the control. Therefore, PMMA does not have independent bacteriostatic activity. Similar results were obtained for the PMMA/Ti-NPs composite material containing 0.001 wt% Ti-NPs. Composite PMMA/Ti-NPs materials containing 0.01–0.1% Ti-NPs exhibit bacteriostatic properties, inhibiting the maximum growth of *E. coli* by 35–40%. At the same time, no “shift” in the lag period was observed.

**Figure 9:**
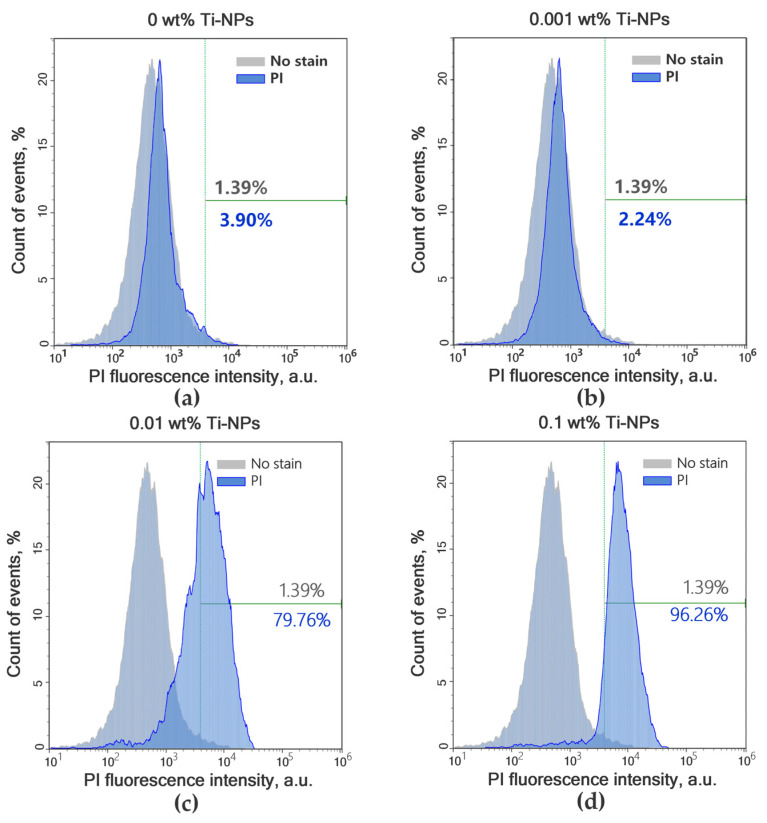
Effect of PMMA/Ti-NPs composite materials on the number of dead (PI-positive) bacterial cells of *E. coli*. Histograms of bacterial cell distribution by geometric mean PI intensity during incubation with PMMA polymer samples containing no Ti-NPs (**a**); during incubation with PMMA/Ti-NPs samples of materials containing 0.001 (**b**), 0.01 (**c**), and 0.1 wt% (**d**) Ti-NPs, respectively.

**Figure 10:**
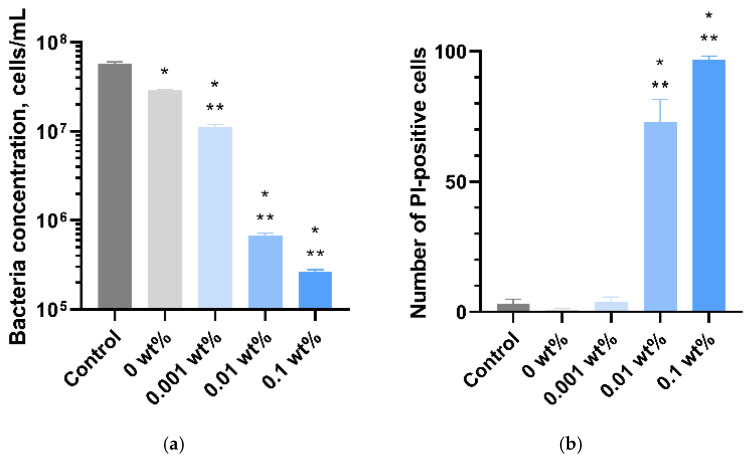
Flow cytometric study of the antibacterial activity of PMMA/Ti-NPs composite materials with different Ti-NPs content: concentration of bacterial cells in suspension in contact with the surfaces of the samples of the studied materials (**a**); number (proportion) of PI-positive (non-viable) bacterial cells *E. coli* cultured in the presence of samples of the plates of the studied materials (**b**). Data are presented as mean values ± SEM (n = 3). *—difference from control (*p* < 0.05), **—relative to samples of material not containing nanoparticles (0 wt%) (*p* < 0.05).

For more accurate analysis of the effect of the obtained materials on the growth and viability of bacterial cells and to identify the bactericidal effect of the studied materials, the flow cytometry method was used, which made it possible to measure the concentration of microorganisms and the proportion of dead (PI-positive) *E. coli* cells after 24 h of incubation with the samples of the plates of the studied PMMA/Ti-NPs composite materials. Figure 9a–d show the diagrams of the intensity distributions of unstained bacterial cells and those stained with PI. The proportion of PI-positive cells in the control group (PMMA without Ti-NPs) was 3.9, while in the experimental groups the proportion of PI cells was 2.24, 79.76, and 96.26% for PMMA/Ti-NPs composite materials containing 0.001, 0.01 and 0.1 wt% Ti-NPs, respectively. To improve the clarity of interpretation of the flow cytometry results, the obtained data are visualized in the form of bar charts in Figure 10a,b.

Figure 10 shows the data on the concentration of live and dead bacterial cells in suspension after 24 h of cultivation with MA and PMMA/Ti-NPs composite materials. PMMA, which does not contain Ti-NPs, had a pronounced bacteriostatic effect and reduced the concentration of live bacteria from ~6.2 × 10^7^ cells/mL to ~3.1 × 10^7^ cells/mL. The PMMA/Ti-NPs material containing 0.001% Ti-NPs had bacteriostatic effect, reducing the concentration of live bacteria to ~1.1 × 10^7^ cells/mL. No reliable bactericidal effect was found for pure PMMA and PMMA/Ti-NPs composite material samples containing 0.001% Ti-NPs. PMMA/Ti-NPs composite materials with Ti-NPs concentrations of 0.01% and 0.1% exerted significant bacteriostatic effect; in the presence of these materials, the concentration of living bacterial cells in the suspension was 2 and 3 orders of magnitude lower than in the control, respectively. The results of assessing the bactericidal effect of the obtained composite materials are shown in Figure 10b. In addition to the bacteriostatic effect, PMMA/Ti-NPs composite materials containing 0.01% and 0.1% Ti-NPs had a pronounced bactericidal effect. After 24 h cultivation of *E. coli* suspension cultures in the presence of these materials, the death of 72.8 ± 8.8% and 96.6 ± 1.5% of the cells was observed for PMMA/Ti-NPs containing 0.01% and 0.1% Ti-NPs, respectively, which was 22 and 30 times higher than the control values (3.3 ± 1.6%).

**Figure 11:**
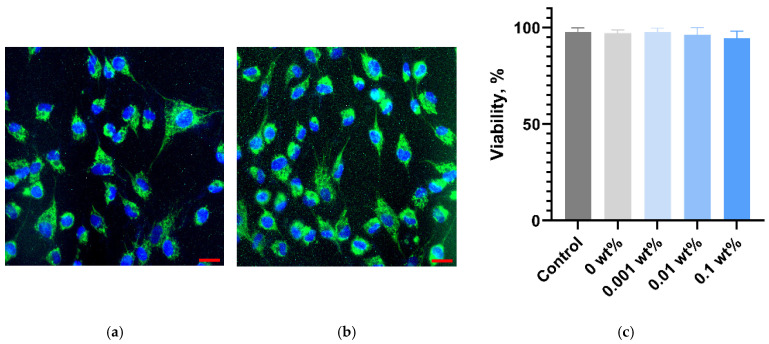
Results of the viability assessment of HSF cell cultures in contact with PMMA/Ti-NPs composite material samples with different Ti-NPs content during 72 h of in vitro cultivation: representative micrographs of the HSF cell culture of the control group (**a**) and the culture cultivated in the presence of PMMA/Ti-NPs composite material containing 0.1 wt% Ti-NPs (**b**); results of the viability assessment of the cell cultures (n = 3) (**c**). The scale bar size in the lower right corner corresponds to 10 µm.

The effect of PMMA/Ti-NPs composite materials on the growth and development of HSF fibroblast cell cultures was also investigated. Microscopic studies showed that the morphology of cells cultured for a long time in the presence of PMMA/Ti-NPs materials did not change, compared to the control cultures (Figure 11a,b). The proportion of viable cells in cultures incubated with all considered samples of PMMA/Ti-NPs composite materials did not change significantly, compared to the control values (97.67 ± 2.21), and amounted to 97.71 ± 1.98, 96.09 ± 3.85 and 94.44 ± 3.72% for PMMA/Ti-NPs materials containing 0.001, 0.01 and 0.1 wt% Ti-NPs, respectively (Figure 11c).

## 4. Discussion

Currently, new approaches to modify photopolymer resins are actively studied in order to obtain structural materials with the necessary physicochemical properties, resistance to external mechanical impact, and significant biological safety [53,54,55]. In a series of previous works, we studied the efficiency of functionalization of polymer matrices of borosiloxane [63], poly(lactide-co-glycolide) [64], and polytetrafluoroethylene [65,66] with metal oxide nanoparticles. In this work, we proposed modification of PMMA-like photopolymer with metallic Ti nanoparticles. There are significant number of works in the literature devoted to modification of polymers with titanium dioxide nanoparticles [67,68]; however, there are practically no works on the efficiency of using Ti-NPs as a functionalizing additive in polymer matrices. To synthesize Ti-NPs, the method of pulsed laser ablation of a titanium metal target in acetone was used. This method is highly efficient and environmentally friendly, allows for high purity of nanoparticles and control of their parameters during the synthesis [66,69]. It was shown that the synthesized nanoparticles had monomodal size distribution (Figure 1a), colloidal solutions were stable (Figure 1b), the UV/Vis optical absorption spectrum corresponded to metallic titanium nanoparticles (Figure 1c), and the nanoparticles had spherical morphology (Figure 1d). Using the developed method, the obtained Ti-NPs dispersions in acetone were added to a photopolymer resin; series of samples were printed from the composite material using MSLA technology, which were standardized rods for mechanical testing, as well as thin plates (Figure 2a,b). Test models of highly detailed porous products were also printed, confirming the suitability of PMMA/Ti-NPs for the design of high-precision products (Figure 2c). The size of the framework walls in the printed model was approximately 500 μm, which is in good agreement with the data reported in recent studies for similar materials [70,71]. Other important properties of printed products made of polymeric materials include resistance to mechanical stress. The literature often reports deterioration of the mechanical properties of polymeric materials at increased concentrations of introduced nanoparticles. The most frequently reported effects include decreased tensile strength [72,73], increased brittleness [74], and decreased bending strength [75]. These negative effects become more pronounced with increasing filler concentrations, are destructive at concentrations ≥ 1% by weight, and may depend on the nature of the introduced nanoparticles [76]. In the context of the mechanical properties of the studied PMMA/Ti-NPs composite materials, an improvement in mechanical properties was observed with the addition of 0.1 wt% Ti-NPs, which was expressed in change in the nature of the material deformation under tension, improvement in their strength and deformation characteristics, compared to the original polymer (Figure 3a,b). The observed behavior of PMMA/Ti-NPs can be due to improved photopolymerization. In this case, the introduction of metal and metal oxide nanoparticles can probably act as co-initiators [77,78]. The data obtained are of great practical importance, since the mechanical characteristics of the material are directly related to its further operational capabilities. It should be noted that unusual mechanical composite polymers and nanosized colloidal systems find practical application, even in such areas as oil production [79], which are far from the average person, which is associated with work at high pressures [80].

Another important characteristic of polymer products obtained in the course of additive manufacturing is the surface topology, including after polishing. As follows from the literature, surface defects are often characteristic of photopolymer composite materials, especially at high filler concentrations. The most common surface defects are cracks, micropores, and microfractures [72]. The surfaces of the plates obtained in the course of the work from the studied composite materials did not have surface defects and had a uniform homogeneous surface (Figure 4a,b), which can have a beneficial effect on the durability and resistance of products from the obtained materials to external factors. As is known, the physicochemical properties of multicomponent systems based on polymers and nanomaterials are directly related to the distribution of particles and the degree of their aggregation in the polymer matrix [81]. In this regard, we assessed the distribution of Ti-NPs in the methacrylate matrix using a modulation interference microscope. The distribution of nanoparticles in the polymer matrix was non-uniform; moreover, the degree of non-uniformity increased with increasing concentration of Ti-NPs (Figure 5a–d). The obtained results are consistent with previously obtained data on other polymer matrices. The presence of specific distribution of the filler in the polymer matrix is due, first of all, to the adhesive properties of the components, their surface energy and rheological behavior of the system [82]. In addition, dispersion methods (ultrasonic treatment, mechanical activation, etc.) play a significant role, determining the degree of particle aggregation and their compatibility with the polymer phase [83]. In general, the study of the distribution of nanoparticles in the photopolymer matrix may be of considerable interest for deeper understanding of the mechanisms of interphase interaction of components and changes in the physicochemical characteristics of the material in order to increase the efficiency and productivity of 3D printing using it [84].

Another common problem with methacrylate materials is the difficulty of achieving high degree of polymerization of the starting components. With incomplete polymerization, the resulting polymer contains monomers that have high activity against living cells and tissues and, as a result, toxicity [85,86]. Thus, one of the important current problems is to reduce the toxicity of methacrylate materials by increasing the degree of polymerization. For this purpose, various polymerization and post-processing modes of polymers are selected [87]. A number of studies have shown that the introduction of nanoparticles can lead to additional cross-linking of polymer molecules, an increase in the depth of UV radiation absorption, and, as consequence, an increase in the degree of polymerization of the matrix [88,89]. In the course of this work, it was shown that the degree of polymerization of the studied polymeric materials functionalized with Ti-NPs was higher than in pure materials. At the same time, the introduction of Ti-NPs did not affect the optical properties of the obtained materials in the visible range (Figure 6a,b). In view of the high popularity of methacrylate photopolymer resins for biomedical applications and as materials for sustainable ecological use, the properties of the studied materials to form biologically active compounds (BAC) and the ability to negatively affect biomolecules are of particular interest. It was shown that PMMA/Ti-NPs composite materials enhanced the generation of hydrogen peroxide and hydroxyl radicals in aqueous solutions (Figure 7a,b). Among various BAC, H_2_O_2_ acts as a relatively stable intermediate product, while ^•^OH are the most reactive oxidants capable of initiating radical reactions [90]. The ability to form free BAC by polymer-based composite materials, especially those based on methacrylates with the addition of nanoparticles, has been very little studied to date. Ti-NPs-functionalized materials induced oxidative damage to DNA and protein molecules in vitro with the formation of 8-oxoguanines in DNA and long-lived reactive forms of proteins (Figure 7c,d). 8-oxoGua is strong endogenous mutagen that causes the replacement of GC pairs with AT pairs in the DNA molecule [91].

Microbiological studies have shown significant inhibitory effects of the synthesized composite materials on suspension bacterial cultures containing Ti-NPs (Figure 8). It is important to note that PMMA/Ti-NPs materials containing increased concentrations of Ti-NPs had significant bactericidal properties, promoting the death of bacterial cells (Figure 9 and Figure 10). A number of other studies demonstrated comparable inhibitory effects in microbiological tests [92,93], but the bactericidal effect of the materials was not detected or studied. The ability of the PMMA/Ti-NPs materials obtained in this work to inhibit the growth and development of bacterial cells is undoubtedly of great practical interest. Despite the fact that titanium is a relatively inert element, its ions (Ti^4+^), in the case of oxidation, can have toxic effect on bacteria, disrupting cellular processes, in connection with which number of studies proposed using Ti-NPs as components for the manufacture of antimicrobial multicomponent materials [94,95]. Photopolymer materials based on methacrylates, including PMMA-like materials, are characterized by high degree of bio- and cytocompatibility, which largely depends on the post-processing of printed products [96,97,98]. All studied samples: both composite materials containing Ti-NPs and pure polymer, did not have significant effect on the growth and development of animal cell cultures in vitro (Figure 11a–c). On the one hand, this is an expected result, since pure titanium is a biocompatible material for mammals, most implants are made of titanium and its alloys [99]. On the other hand, it is known that nanoparticles can be toxic due to their size, regardless of their chemical structure. So, it is known that some preparations of titanium nanoparticles can be toxic [100]. In this work, non-toxic titanium nanoparticles for mammalian cells were obtained using laser ablation. The absence of cytotoxic effect, despite the pronounced antimicrobial activity, makes the obtained PMMA/Ti-NPs composite materials promising for biomedical applications that require combination of antiseptic properties and safety for mammalian cells. Thus, our composite materials based on polymethacrylates modified with Ti-NPs may be of considerable interest due to their environmental safety, optimal mechanical and physicochemical properties, promising antibacterial activity and the absence of pronounced toxic effect on animal cells in vitro.

## 5. Conclusions

Thus, a simple method for obtaining composite materials based on transparent polymethyl methacrylate-like photopolymer resin and metallic titanium nanoparticles is proposed. Products with accuracy of about 50 μm can be printed from the obtained modified photopolymer resins using MSLA technology. It is shown that the addition of nanoparticles reduces the fragility of products, while more complete photopolymerization of the resin is observed. The addition of nanoparticles does not affect the surface topology and polishability of products. Composite materials in water when heated can produce ROS, which probably leads to damage to biopolymers. Composite materials inhibit the growth of *E. coli* bacterial cells. Bactericidal action of the surfaces of the obtained materials is shown. Despite significant antibacterial properties of the synthesized materials, no negative effect on the growth and development of adhesive cultures of eukaryotic cells in vitro was found. In general, promising materials for biomedical applications were obtained.

## Data Availability

The raw data supporting the conclusions of this article will be made available by the authors on request.

## Abbreviations

The following abbreviations are used in this manuscript:

PMMA	Polymethyl methacrylate
Ti-NPs	Titanium nanoparticles
H_2_O_2_	Hydrogen peroxide
^•^OH	Hydroxyl radical
PI	Propidium iodide
MIM	Modulation interference microscopy
MSLA	Masked Stereolithography
ROS	Reactive oxygen species
